# Diverse, Culturally Rich Approaches to Family Care in the United States

**DOI:** 10.1093/geroni/igab046.1033

**Published:** 2021-12-17

**Authors:** Manka Nkimbeng, Lauren Parker

**Affiliations:** 1 University of Minnesota, Minneapolis, Minnesota, United States; 2 Johns Hopkins Bloomberg School of Public Health, Baltimore, Maryland, United States

## Abstract

Despite the projected rise in the diversity of caregivers and caregiving in the US, the health system is not prepared to accommodate this growth. Interventions and supports often are not adequately tailored to meet the cultural needs of older adults. Additionally, the limited interventions available for racial/ethnic minority populations frequently fail to capture and report culturally tailored perspectives. Therefore, the purpose of this presentation is to describe how culture influences caregiving in the US. Specifically, it will: (1) provide a contemporary definition of culture; (2) identify cultural domains that impact caregiving; (3) offer examples of how caregiving is influenced by different cultural/demographic backgrounds; (4) provide examples of culturally tailored caregiving programs, and (5) discuss how to approach cultural needs that may not be addressed by current interventions.

